# Hand-Book of Ophthalmology

**Published:** 1878-08

**Authors:** 


HAND-BOOK OF OPHTHALMOLOGY. By Professor C. Schweizzer, of the University of Berlin. Translated from the third German edition by Porter Farley, M.D., Rochester, New York, with diagrams and other illustrations. Philadelphia: J. B. Lippincott & Co. 1878.

This book of 550 pages seems to be an exhaustive treatise on diseases of the eye, and will doubtless prove a valuable addition to the libraries of ophthalmologists.

